# David Williams, the Queer Philanthropist

**Published:** 1920-04-10

**Authors:** 


### David Williams, the Queer Philanthropist.

There are several reasons why hospital men should take an interest in David Williams, who founded what has now become the Royal Literary Fund. He was a strange pioneer of philanthropy, and almost the only sympathisers whom Williams found at the start were doctors. Again, hospitals rely so much upon the power of the pen and the help of the theatre that they may well sympathise with a fund in aid of authors in distress. In fact, hospitals and the Royal Literary Fund are both concerned in the fate of the same person, since Dr. Alexander Johnson, M.D., when presiding at a public meeting in 1790, the year of the foundation of the Fund, said that its object was to "support deserving authors in *sickness*, distress, old age, and at the termination of life, and to give temporary relief to the widows and orphans of those who have any claims on the public from having written any useful book." Little is generally known about the fund, except from the brilliant speeches which have usually adorned the anniversary dinners; and the Royal Literary Fund cannot make play with the "cases" which it helps. It would defeat its own object and hinder applications if it did not act the part of a confidential friend to those in need. The fund must proceed by other measures, and has done well to entrust to Mr. E. V. Lucas the brief biography of David Williams, which has been issued by Mr. Murray at half-a-crown.

David Williams himself was one of the last of the eighteenth-century philanthropists, and busied himself, after the manner of Rousseau, with speculation, methods of education, and "the universal principles of religion and morality." Philanthropists are a queer class of people, and David Williams with his Methodist ministry, preoccupation with women, delightful school, political adventures in Paris during the Revolution, and marvellous persistence in founding the Literary Fund in spite of every discouragement, is an attractive and often admirable figure. Mr. Lucas might well return to the subject and give us something more than this sketch. But his space was limited, and more than half the little work is filled with extracts from the speeches made at the dinners of the fund. Thackeray, Trollope, Disraeli, Gladstone, Livingstone, Manning, in turn, are quoted, and one of the best passages is from the speech of King Edward VII. We are all in the debt of literature, and can acknowledge it in two ways. The first is to buy books, and not merely to be a parasite at the mercy of the lending library. The second is to support the Royal Literary Fund, which, for the reason stated above, is perhaps the most difficultly placed of all charities.

